# Absolute quantification reveals the stable transmission of a high copy number variant linked to autoinflammatory disease

**DOI:** 10.1186/s12864-016-2619-0

**Published:** 2016-04-23

**Authors:** M. Olsson, M. Kierczak, Å. Karlsson, J. Jabłońska, P. Leegwater, M. Koltookian, J. Abadie, C. Dufaure De Citres, A. Thomas, Å. Hedhammar, L. Tintle, K. Lindblad-Toh, J. R. S. Meadows

**Affiliations:** Department of Medicine, Rheumatology Unit, Karolinska Institute, Stockholm, Sweden; Department of Medical Biochemistry and Microbiology, Science for Life Laboratory, Uppsala University, Uppsala, Sweden; Department of Clinical Sciences of Companion Animals, Utrecht University, Utrecht, Netherlands; Broad Institute of MIT and Harvard, Boston, MA USA; LUNAM University, Oniris, AMaROC Unit, Nantes, F-44307 France; ANTAGENE Animal Genetics Laboratory, La Tour de Salvagny, Lyon 69 France; Department of Clinical Sciences, Swedish University of Agricultural Sciences, Uppsala, Sweden; Wurtsboro Veterinary Clinic, Wurtsboro, New York USA

**Keywords:** Copy number variation, Autoinflammation, Droplet digital PCR, Quantitative PCR

## Abstract

**Background:**

Dissecting the role copy number variants (CNVs) play in disease pathogenesis is directly reliant on accurate methods for quantification. The Shar-Pei dog breed is predisposed to a complex autoinflammatory disease with numerous clinical manifestations. One such sign, recurrent fever, was previously shown to be significantly associated with a novel, but unstable CNV (CNV_16.1). Droplet digital PCR (ddPCR) offers a new mechanism for CNV detection via absolute quantification with the promise of added precision and reliability. The aim of this study was to evaluate ddPCR in relation to quantitative PCR (qPCR) and to assess the suitability of the favoured method as a genetic test for Shar-Pei Autoinflammatory Disease (SPAID).

**Results:**

One hundred and ninety-six individuals were assayed using both PCR methods at two CNV positions (CNV_14.3 and CNV_16.1). The digital method revealed a striking result. The CNVs did not follow a continuum of alleles as previously reported, rather the alleles were stable and pedigree analysis showed they adhered to Mendelian segregation. Subsequent analysis of ddPCR case/control data confirmed that both CNVs remained significantly associated with the subphenotype of fever, but also to the encompassing SPAID complex (*p* < 0.001). In addition, harbouring CNV_16.1 allele five (CNV_16.1|5) resulted in a four-fold increase in the odds for SPAID (*p* < 0.001). The inclusion of a genetic marker for CNV_16.1 in a genome-wide association test revealed that this variant explained 9.7 % of genetic variance and 25.8 % of the additive genetic heritability of this autoinflammatory disease.

**Conclusions:**

This data shows the utility of the ddPCR method to resolve cryptic copy number inheritance patterns and so open avenues of genetic testing. In its current form, the ddPCR test presented here could be used in canine breeding to reduce the number of homozygote CNV_16.1|5 individuals and thereby to reduce the prevalence of disease in this breed.

**Electronic supplementary material:**

The online version of this article (doi:10.1186/s12864-016-2619-0) contains supplementary material, which is available to authorized users.

## Background

The role copy number variants (CNVs) play in genomic processes spanning from evolution [1–4], through population genetic diversity [5–8] and disease susceptibility [9, 10] is under constant investigation. Whilst CNVs are less common than single nucleotide polymorphisms (SNPs), these genomic gains and losses are more likely to have an impact on phenotypic diversity [9]. One of the key mechanisms of action for CNVs is through the disruption of gene or regulatory element dosage, leading to perturbed gene expression. In order to dissect the impact these structural variants play, it is essential that they be accurately detected and quantified. Methods such as fluorescence in situ hybridisation (FISH), multiplex ligation-dependent probe amplification (MLPA) and array comparative genomic hybridisation (aCGH) can all be used to detect copy number variants, but the establishment of mode of transmission or genotype may fall to alternate PCR based methods such as quantitative PCR (qPCR), or more recently, droplet digital PCR (ddPCR).

The detection of canine CNVs has advanced dramatically from the first array CGH which revealed 155 variants [11], through to a catalogue of more than 1,600 polymorphisms which have been assayed in both domestic dogs and a variety of members from the Canidae family [5–8]. Through these studies and others (summarised in [12]), we have gained insight into what sets domestic dogs apart from their wild ancestors (e.g. a CNV gain in a region encompassing *AMY2B* drives increased amylase activity and the ability for domestic animals to digest a starch rich diet [1]) and an understanding of dog phenotypic diversity and disease susceptibility (e.g. a copy number gain encompassing *FGF3*, *FGF4* and *FGF19* which results in the breed defining dorsal ridge of the Rhodesian and Thai Ridgeback, but also predisposes these breeds to dermoid sinus [13]). The link between genotype and phenotype relies not only on the ability to identify CNVs, but also on the accuracy of variant quantification. If the CNV is to be used diagnostically, the importance of the latter cannot be overestimated.

This study focuses on two breed specific CNVs found in the Chinese Shar-Pei; the “traditional” (14.3 kb) variant and the “meatmouth” (16.1 kb) variant, located in an overlapping region of chromosome 13 [14]. It has been reported that a high copy number of the 16.1 kb variant is associated with the increased expression of *Hyaluronan Synthase 2* (*HAS2*), the driver of long-chain hyaluronan (HA) synthesis [14, 15]. The elevated expression of *HAS2* results in cutaneous hyaluronosis, creating the Shar-Pei’s distinctive thickened and folded skin [16–18]. The increased copy number of the 16.1 kb variant was also shown to be significantly correlated to the occurrence of a breed specific fever syndrome [14]. It was hypothesised that the recurring fever followed the cyclical over production and subsequent degradation of HA and that low molecular weight HA was acting as a danger associated molecular pattern (DAMP) triggering the release of inflammatory interleukins [14, 19].

More recently it was reported that this fever syndrome was actually one of a spectrum of clinical signs including arthritis, ear (otitis) and skin (vesicular hyaluronosis) inflammation and amyloidosis that were encompassed by the larger Shar-Pei Autoinflammatory Disease (SPAID) [20]. In a genetic study which utilised 250 carefully phenotyped individuals, genome wide association analyses showed that the five clinical signs of SPAID overlapped one genetic locus; a region which also encompassed *HAS2* and both the 14.3 kb and the 16.1 kb copy number variants [20].

When the 14.3 kb and the 16.1 kb CNVs were first measured, the methodology employed was quantitative PCR (qPCR) [14]. This was a primer-limited multiplex qPCR where the result of an unknown individual was calibrated to the result of an individual with known diploid copy number (i.e. CNV = 2). The error in this measurement was therefore a composite of the errors from four PCR reactions (two individuals for both a target CNV and reference PCR) and quadruplet replications. When applied to the 16.1 kb variant this resulted in a continuum of copy number estimates, from two to fifteen or more [14]. An alternative to qPCR is droplet digital PCR (ddPCR). In the latter case, restriction digested DNA and a multiplex PCR mix are evenly partitioned across many thousands of oil droplets. After the completion of thermocycling, the initial concentration of template DNA is determined from the Poisson distribution of positive (those containing amplified target, be it CNV or reference) and negative (those containing no amplified target) reaction droplets [21]. Results comparing the two PCR methodologies have generally shown gains in precision and reproducibility when using ddPCR versus qPCR [22–24] although, as noted by others, this can come at an increased cost per reaction [25].

With the recent advancement in defining Shar-Pei Autoinflammatory Disease and the availability of alternate methods of quantifying copy number variants, the aims of the current study were three-fold, i) to identify a reliable CNV measurement method (quantitative PCR versus droplet digital PCR), ii) to apply that method to investigate the relationship between CNV load, the occurrence of SPAID and SPAID sub-phenotypes and iii) to assess the utility of CNV variants as a genetic test for SPAID.

## Results

### Droplet digital PCR has reduced variability compared to quantitative PCR

**Fig. 1:**
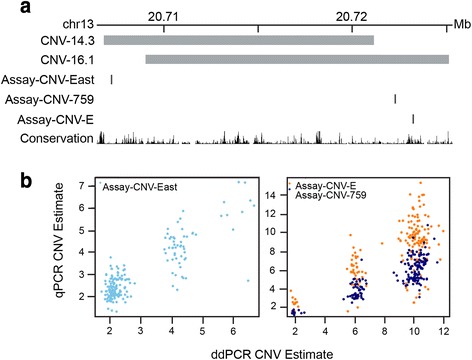
**a** The genomic location of the two CNVs and **b** the comparison of results obtained using quantitative PCR (qPCR) and droplet digital PCR (ddPCR) for assays in each (CNV_14.3: Assay-CNV-East; CNV_16.1: Assay-CNV-759 and Assay-CNV-E). Regression analysis showed Assay-CNV-East reported reasonably similar results (*r* ^*2*^ = 0.72), whilst concordance was lower for Assay-CNV-759 (*r* ^*2*^ = 0.64) and Assay-CNV-E (*r* ^*2*^ = 0.44). In each assay it can be seen ddPCR reports tighter clustering at higher qPCR values

Copy numbers were measured for 196 Shar-Pei (Additional file 1: Table S1) with three assays (Assay-CNV-East, Assay-CNV-759 and Assay-CNV-E, Fig. 1a) and two methodologies, droplet digital PCR (ddPCR) and quantitative PCR (qPCR). The relationship between assays and methodologies is illustrated in Fig. 1. The linear correlation between methodologies was found to be the highest for CNV-East (*r*^*2*^ = 0.72, Fig. 1b), followed by CNV-759 (*r*^*2*^ = 0.64) and was the lowest for CNV-E (*r*^*2*^ = 0.44). The value for CNV_14.3 as measured by Assay-CNV-East was on average 0.14 CNVs smaller when measured by ddPCR than qPCR. For CNV_16.1 the pattern was discordant with CNV measures on average 3.44 and 0.77 larger (Assay-CNV-759 and Assay-CNV-E respectively), when measured with ddPCR compared to qPCR. Whilst qPCR CNV measures for one individual were not always in agreement between Assay-CNV-759 and Assay-CNV-E (Average CNV difference = 2.66 ± 2.60), they were in extremely close agreement when measured with ddPCR (Average CNV difference = 0.01 ± 0.52). Examples of these inconsistencies in qPCR compared to ddPCR at an individual level are illustrated in Additional file 2: Figure S1. From this point forward, the results of Assay-CNV-759 and Assay-CNV-E will be presented as the result for CNV_16.1, as the former were interchangeable.

### Breed specific CNVs are stable and inherited in a bi-allelic fashion

**Fig. 2:**
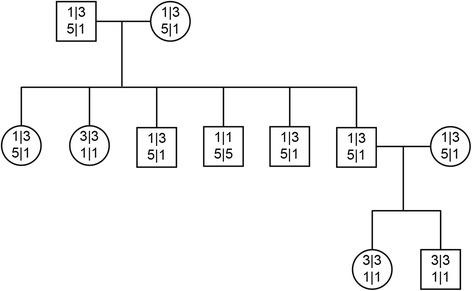
An example pedigree illustrates the segregation of CNV alleles across generations. The second generation shows a distribution that is in keeping with Mendelian segregation. CNV results are coded to reflect the number of alleles per chromosome. Using the male in generation one as an example, the result for CNV_14.3 (copy number: 4; alleles: 1|3) is written above that for CNV_16.1 (copy number: 6; alleles: 5|1)

ddPCR showed clearly that the results for both CNV_14.3 and CNV_16.1 formed three clusters (CNV_14.3: 2, 4, 6; CNV_16.1: 2, 6, 10) suggesting that these copy number variants may be acting as stably inherited alleles. We tested this hypothesis in extended pedigrees (*n* = 92, Fig. 2; Additional file 1: Table S1; Additional file 2: Figure S2) and found that this was in fact true. CNV_14.3 was demonstrated to have two alleles comprising either 1 or 3 copies and for CNV_16.1, alleles of 1 or 5 copies were shown. For the full set of individuals included in the study (*n* = 327), no alternate combinations were observed.

A pattern of CNV genotype segregation was observed whereby CNV_14.3 = 2/CNV_16.1 = 10 accounted for 64.5 % of the CNV pairs, followed by CNV_14.3 = 4/CNV_16.1 = 6 (30.3 %) and CNV_14.3 = 6/CNV_16.1 = 2 (4.3 %). However we also recorded the following pairs, CNV_14.3 = 4/CNV_16.1 = 2 (0.6 %) and CNV_14.3 = 2/CNV_16.1 = 6 (0.03 %).

### CNV_16.1 is associated with increased *HAS2* expression and SPAID risk

Olsson et al., [14] showed that the expression of genes *HAS2* and *HASas* increased with increasing CNV_16.1 copy number. This experiment was replicated using their gene expression measures and new ddPCR calculations of copy number for the assayed fibroblast DNA. The same trend of increased gene expression with CNV_16.1 copy number was observed (Additional file 2: Figure S3).

The ddPCR method provided the resolution required to demonstrate that both duplications were stably transmitted. In order to assess disease correlation, phenotype positive and negative genotype and allele counts were made (Table 1), and 2x2 allele risk- and odds- ratio calculations performed (Table 2). As reported above, 64.5 % of duplication pairs form the pattern of CNV_16.1 = 10/CNV_14.3 = 2. This was patterned carried forward to Table 1, where a high proportion both CNV_16.1 = 10 and CNV_14.3 = 2 were recorded in affected individuals in each disease set. It was shown previously that CNV_14.3 and CNV_16.1 are breed specific variants, and that a copy number of two is observed at both genomic locations in all other breeds [14]. We therefore assessed the link between CNV_16.1 and disease, and not CNV_14.3.

**Table 1:** Genotype and allele counts for the age limited SPAID cohort and each subphenotype therein^a^

		SPAID^b^		Fever		Arthritis		Vesicular Hyaluronosis		Otitis		Amyloidosis^c^	
		+	-	+	-	+	-	+	-	+	-	+	-
	Genotype												
CNV_14.3	2	128	15	93	30	62	28	35	36	31	43	28	13
	4	24	17	20	19	17	15	9	19	2	25	2	4
	6	3	2	1	2	0	3	0	3	1	3	1	0
CNV_16.1	2	3	3	1	3	0	4	0	4	1	4	1	0
	6	25	16	21	18	17	15	9	19	2	25	3	4
	10	127	15	92	30	62	27	35	35	31	42	27	13
	Allele												
CNV_14.3	1	280	47	206	79	141	71	79	91	64	111	58	30
	3	30	21	22	23	17	21	9	25	4	31	4	4
CNV_16.1	1	31	22	23	24	17	23	9	27	4	33	5	4
	5	279	46	205	78	141	69	79	89	64	109	57	30

^a^The proportion of each cohort (affected/unaffected) was, SPAID (155/34), Fever (114/51), Arthritis (79/46), Vesicular Hyaluronosis (44/58), Otitis (34/71). ^b^SPAID negative is equivalent to C1. ^c^Amyloidosis (31/17) is not age limited, rather a negative result was determined by post-mortem histopathology

**Table 2:** Risk- and odds-ratio test results for the disease associated allele (CNV_16.1, allele 5) and control group C1

Risk Allele		SPAID		Fever		Arthritis		Vesicular Hyaluronosis		Otitis		Amyloidosis^a^	
CNV_16.1|5	Risk	1.31	1.17–1.46	1.33	1.12–1.58	1.32	1.11–1.57	1.33	1.11–1.59	1.39	1.17–1.66	1.04	0.90–1.20
	Odds	4.10	2.48–6.74	4.26	2.19–8.30	3.97	1.94–8.11	4.20	1.78–9.89	7.65	2.47–23.71	1.52	0.38–6.09
	*p*-value	<0.0001		<0.0001		0.0002		0.0010		0.0001		0.7163	

^a^C3 and not C1 (SPAID negative) allele counts were used for the comparison to the Amyloidosis subphenotype as C1 individuals were not all assessed for the absence of amyloid deposits in kidney tissues. Fisher two tailed exact probability test was used to calculate significance using allele counts from Table 1

As reported in Table 1, the CNV_16.1 allele 5 (CNV_16.1|5) is the variant highly significantly associated to SPAID (*p* < 0.0001), and confered a greater than four fold increase in odds-ratio (Table 2). Similar odds ratios were noted for four of the five phenotypes of SPAID, but not for Amyloidosis.

### CNV_16.1 is predictive of disease risk

The utility of CNV_16.1 and CNV_14.3 in a genetic test for SPAID was evaluated using random forest (RF) [26] and J48 decision trees [27]. Given the low membership of C1 (*n* = 34), a comparison with C2 (*n* = 80) was also calculated. The best model was generated using the data set containing the allelic information from both CNVs and the maximum number of participants (C2 RF AUC = 0.613, Table 3), although the performance of all models compared using the C2 control group were similar (maximum AUC difference 0.014).

**Table 3:** The comparison of two classification models of SPAID using alternate control groups^a^

Genotypes from marker(s)	Control group	Tree	TP	FP	Precision	Recall	F-measure	AUC
CNV_14.3 and CNV_16.1	C2	J48	0.715	0.389	0.706	0.715	0.708	0.605
		RF	0.715	0.389	0.706	0.715	0.708	0.613
	C1	J48	0.810	0.822	0.671	0.810	0.734	0.575
		RF	0.810	0.822	0.671	0.810	0.734	0.575
CNV_14.3	C2	J48	0.715	0.389	0.706	0.715	0.708	0.605
		RF	0.715	0.389	0.706	0.715	0.708	0.611
	C1	J48	0.820	0.820	0.673	0.820	0.739	0.457
		RF	0.820	0.820	0.673	0.820	0.739	0.575
CNV_16.1	C2	J48	0.681	0.473	0.661	0.681	0.661	0.599
		RF	0.711	0.391	0.702	0.711	0.704	0.612
	C1	J48	0.820	0.820	0.673	0.820	0.739	0.457
		RF	0.810	0.822	0.671	0.810	0.734	0.580

^a^Two control groups were considered. Control 2 (C2, *n* = 80) contained all dogs free from SPAID, irrespective of age, whilst Control 1 (C1, *n* = 34) included only those individuals older than 60 months with no signs of SPAID. The counts required to calculate the receiver operator curve (ROC) area are reported, including true positive (TP) and false positive (FP)

### Local genomic architecture may explain lower than expected AUC

In the genome wide association analysis (GWAS) of SPAID, Olsson et al. [20] noted high linkage disequilibrium (LD, *r*^*2*^ > 0.8) across the genomic regions associated with all five SPAID sub-phenotypes. A GWAS was repeated with the addition of CNV_16.1 as a biallelic marker to the genome wide set. With our reduced cohort (SPAID, *n* = 155; C1, *n* = 34) and the same methodology as reported previously [20], we found that whilst not reaching genome-wide significance (λ = 1.04; *p*_CNV_16.1_ = 2.78 × 10^-5^; *p*_Bonferroni_ = 4.55 × 10^-7^), the CNV marker explained approximately 10 % of the genetic variance (CNV_16.1 = 9.7 %) and close to 25 % of the genetic heritability (CNV_16.1 = 25.8 %).

## Discussion

We assessed the ability of two PCR methods, quantitative PCR (qPCR) and droplet digital PCR (ddPCR), to accurately quantify two breed specific CNVs in relation to the susceptibility of Shar-Pei Autoinflammatory Disease (SPAID). We found that whilst disease association remained highly significant whether the CNV assays were measured with either qPCR or ddPCR (All assays, either control group; Mann Whitney *p*-value < 0.001), only ddPCR allowed for the true bi-allelic pattern of inheritance to be followed. In fact, ddPCR also revealed that whilst CNV pairs were typically inherited in a predictable manner, e.g. CNV_14.3 = 2 copies with CNV_16.1 = 10 copies), recombination does occur at this part of the genome and other combinations of results are observable, albeit at a much lower frequency. The continuum of CNV values we observed when using the qPCR method has also been noted by others using similar means [28]. It is likely that their ability to resolve copy number alleles would also improve with ddPCR. This point is extremely important in the context of genetic testing and future breeding programs.

The reasons as to why ddPCR was able to more clearly resolve copy number in this setting may simply be due to the mechanics of that method versus qPCR. For example, in our hands and with a subset of 50 samples, we generated and read approximately 13,000 droplets per individual tested. Of those accepted droplets, 6,000 on average contained the VIC labelled reference PCR product which equates to 6,000 separate C7orf28b 90 bp PCR products (Additional file 2: Table S3). This is in comparison to the four PCR replicates that were measured for qPCR. The number of FAM droplets is dependent on the number of 16.1 kb or 14.3 kb segments and so averaging this value was not appropriate. There are also differences in the way the two methods estimate errors and also CNV results, e.g. the normalisation to a reference individual for the delta delta CT method of qPCR versus absolute concentration calculation of ddPCR [21].

However, the differences in copy number results (Fig. 1b) may also be a reflection of the architecture in surveyed genome region. It is known that PCR can be more challenging in GC rich regions, but this does not seem to be a contributing factor in this case. Both Shar-Pei copy number elements, and the 100 kb region encompassing them, are estimated to slightly less than the average GC content for dog and a set of primates (36 % versus 41 % in dog, 43 % in human and 42 % in chimpanzee and macaque [29]). It seems more likely that there are complex secondary structure issues that are resolved when the target genomic DNA is digested into small five kb blocks as part of the ddPCR protocol. This may facilitate easier primer binding and product elongation.

Shar-Pei Autoinflammatory Disease (SPAID) is both a phenotypically and genetically complex condition [20]. The 16.1 kb CNV is the marker most associated with disease, explaining 25 % of the genetic heritability in the studied population, however it is the interplay between this genomic region and as yet other undiscovered genes, plus the effect of the dog’s environment, that ultimately determines that individual’s clinical disease status.

In summary, carrying the CNV_16.1|5 allele will increase a dog’s odds of developing disease by four-fold (Table 2), but this predictive measure does not mean that the same carrier will present with clinical disease, be it fever, arthritis, otitis, vesicular hyaluronosis or amyloidosis. For that reason, we would suggest that the results of the CNV_16.1 measurement be used to inform mating strategies, preferentially breeding a homozygous CNV_16.1|5 individual (i.e. CNV_16.1 = 10 copies) with either a CNV_16.1 heterozygote (i.e. CNV_16.1 = 6 copies) or CNV_16.1|1 homozygote (i.e. CNV_16.1 = 2 copies). However, there appear to be very few CNV_16.1|1 homozygotes (CNV_16.1 = 2 copies) in the general population; we found only 16 within our tested set of 327 Shar-Pei. Whilst it may seem prudent to use these individuals widely in order to quickly reduce the number of homozygous CNV_16.1|5 dogs, this may have dire results for the breed. The overuse of CNV_16.1|1 homozygotes could serve to reduce the overall genetic diversity of the breed and perhaps even enrich for as yet unknown diseases.

## Conclusions

These results clearly illustrate the potential for ddPCR to quantify the true count of alleles at CNVs. This gain of precision revealed a previously unknown pattern of allele segregation for two Shar-Pei Specific CNVs and in doing so allowed for the evaluation of a genetic test. This test could now be used in carefully managed breeding programs to methodically reduce the number of individuals carrying the disease associate allele (CNV_16.1|5) without dramatically reducing the breed’s overall genetic variation.

## Method

### SPAID phenotype characterisation

Purebred pet Shar-Pei were sampled from France, the Netherlands, Sweden and the United States following owner consent and ethical approvals (See Declaration). Owners submitted a standardised questionnaire regarding the overall health of their animal. Where possible they also provided detailed medical records and pedigree information. This information was compiled, and in conjunction with veterinarians, used to determine an individual’s case or control status. As per Olsson et al. [20], cases were defined based on the clinical signs of SPAID. These were, SPAID (S, *n* = 155): Any one or more of the five inflammatory signs of SPAID; Fever (F, *n* =114): Recurrent bouts of fever lasting 6–72 h with no underlying infection; Arthritis (Ar, *n* = 79): Recurrent or prolonged bouts of joint (hock) inflammation with no known underlying infection; Vesicular Hyaluronosis (V, *n* = 44): Dermatological vesicular changes to the skin leading to recurrent or persistent secondary inflammation; Otitis (O, *n* = 34): Recurrent or chronic inflammation of the ears; Amyloidosis (Am, *n* = 31): Congo Red stained amyloid deposits observed in a post mortem kidney biopsy. These categories were not discrete (Additional file 2: Table S2).

Three control groups were defined. The first, Control 1 (C1, *n* = 34), encompassed individuals older than 60 months with no signs of SPAID, whilst Control 2 (C2, *n* = 80) was more relaxed and contained all dogs free from SPAID, irrespective of age. Control 3 (C3, *n* = 17) was specific for the sub-phenotype of Amyloidosis and included only those healthy individuals that were negative for Congo Red stained amyloid deposits in post mortem kidney tissue and were also free from a clinical history of unexplained inflammation. Additional pedigree material (*n* = 92, Additional file 1: Table S1) was used to test the stability and transmission of both CNVs assayed.

### Genotyping and genetic analysis

Two breed-specific copy number variants were identified in Olsson et al., [14]. These were termed Traditional (14.3 kb; CanFam3.1 chr13:20,706,841-20,721,149) and Meatmouth (16.1 kb; CanFam3.1 chr13: 20,709,024-20,725,124). To aid clarity, in this manuscript they are named CNV_14.3 and CNV_16.1 to reflect their length as opposed to Shar-Pei breed subtypes.

Olsson et al., [14] designed two assay sets (primer pair and fluorescently labelled probe) that are also utilised in the current analysis. The first was unique to CNV_16.1, Assay-CNV-E, and the second acts as a housekeeper for normalisation, Assay-C7orf28b. Two new assay pairs were designed for the current work. One set was designed to a unique region of CNV_14.3, Assay-CNV-East, and the other is an additional set for CNV_16.1, Assay-CNV-759. All primer and probe assay sets are listed in Additional file 2: Table S3 and illustrated in Fig. 1.

We utilised two methodologies, quantitative real-time PCR (qPCR) and droplet digital PCR (ddPCR) to estimate the number of CNV_14.3 and CNV_16.1 copies present in each DNA sample tested. For qPCR, the (ΔΔCT) relative quantification method and a reference individual with known copy number status (German Shepherd 95, GSP95, Additional file 1: Table S1) was used. The multiplex reaction contained a primer limited target copy number assay for one of the following target assays, Assay-CNV-East or Assay-CNV-759 or Assay-CNV-E, plus the housekeeper assay, Assay-C7orf28b. A target assay comprised 300nM of forward and reverse primer plus 250nM of FAM labelled probe (LifeTechnologies) whilst the housekeeper contained 900nM of forward and reverse primer plus 250nM of VIC labelled probe (LifeTechnologies). These multiplex reactions were performed in quadruplet using 10 ng of gDNA, Genotyping Master Mix (LifeTechnologies) and a 7900HT Real-Time PCR machine (LifeTechnologies) following manufacturers specifications.

For ddPCR, absolute quantification was performed using 15 ng of *Dra*I (NEB) pre-restriction digested DNA in a 20ul reaction mix containing 1x ddPCR Supermix for Probes (BioRad) with 900nM target and reference primers and 250nM of target and reference probes. The primers and probes were the same used for qPCR (Additional file 2: Table S3). The ddPCR reaction mix was portioned into oil droplets following the manufacturer’s (BioRad) specifications [30], amplified at 58 °C in a C1000 Touch thermocycler (BioRad) and quantified using a QX100 instrument (BioRad). QuantaSoft v1.3.1.0 was used for the visualisation of digital droplet results and for the calculation of template per droplet based on a Poisson distribution.

### Statistical analysis

In order to assess the utility of either Assay-CNV-E or Assay-CNV-759 as a diagnostic test for SPAID, we used the allelic values of each assay as variables and constructed predictive models using WEKA software [27]. We built the models using two different statistical-learning algorithms: J48 implementation [27] of Quinlan’s [31] C4.5 decision trees and Breiman’s [26] Random Forest (RF). The two models were selected to overcome any biases in classifier choice. RF-based classifiers have proven robust and reliable on most types of data and as such have become the primary algorithm of choice. RFs, being an ensemble method, provide only a limited insight into the actual classification process in their basic version. This limits our ability to obtain additional insight into the nature of the modelled phenomenon. For this reason we also constructed an extra set of classifiers using another popular and robust statistical-learning algorithm, decision trees. Both types of classifiers were evaluated in a standard 10-fold cross-validation.

### Genome wide association study

Bialellic results from the ddPCR CNV_16.1 assay were combined with existing Illumina CanineHD array data [20] for the available 155 SPAID cases and 34 control individuals. GenABEL v1.7-2 [32] in R v2.15.0 was used to perform the analysis. Quality control involved tests for missing genotype calls for single SNP and individuals (**<**5 %), minor allele frequency (<0.05) and strong deviations from Hardy-Weinberg Equilibrium (HWE, *p* > 1 x10^-8^). A FDR rate of 0.2 was applied to the controls. From the starting set of 173,663 markers genotyped, 109,966 remained for analysis. To correct for population stratification, a polygenic mixed model was fitted which encompassed the Identity-By-State matrix.

### Ethics statement

Samples were collected following owner consent and with the following ethical approvals: DEC Utrecht University Ethical Committee, permit #10813; Ethical Board for Experimental Animals in Uppsala, permit #C103/10; Massachusetts Institute of Technology Committee for Animal Care, permit #0910-074-13.

### Availability of data and material

The dataset supporting the conclusions of this article is included within the article (and its additional files).

## Additional files

Additional file 1: Table S1. Summary information for each individual included in the current study. (XLSX 69 kb) Additional file 2: Table S2. Phenotypic overlap observed within the genetic test cohort. **Table S3.** Primers and Probes used in analysis. **Figure S1.** Illustrative plots for two individuals showing the results obtained for each assay and methodology. **Figure S2.** Five pedigrees illustrating the segregation of copy number variant (CNV) alleles. **Figure S3.** The relationship of increased HAS2 and HAS2as gene expression with increased CNV_16.1 copy number holds with droplet digital PCR (ddPCR) CNV measures. (DOCX 1628 kb)

## Abbreviations

aCGH: array comparative genomic hybridisation
*AMY2B*: *amylase, alpha 2B*
AUC: area under the curve
CNV: copy number variant
DAMP: danger associated molecular pattern
ddPCR: droplet digital polymerase chain reaction
FDR: False discovery rate
*FGF3, FGF4* and *FGF19*: *Fibroblast Growth Factor 3*, *4* and *19*
FISH: fluorescence in situ hybridisation
GWAS: genome wide association analysis
HA: hyaluronan
*HAS2*: *Hyaluronan Synthase 2*
HWE: Hardy-Weinberg Equilibrium
kb: kilobase
LD: linkage disequilibrium
MLPA: multiplex ligation-dependent probe amplification
nM: nanomolar
PCR: polymerase chain reaction
qPCR: quantitative polymerase chain reaction
RF: random forest
SNP: single nucleotide polymorphisms
SPAID: Shar-Pei Autoinflammatory Disease
