# Supplementary material for: Absolute quantification reveals the stable transmission of a high copy number variant linked to autoinflammatory disease
Source: BMC Genomics. 2016 Apr 23;17:299. doi: 10.1186/s12864-016-2619-0 (PMC4841964; doi:10.1186/s12864-016-2619-0)
Supplement: Additional file 2: Table S2. — Phenotypic overlap observed within the genetic test cohort. Table S3. Primers and Probes used in analysis. Figure S1. Illustrative plots for two individuals showing the results obtained for each assay and methodology. Figure S2. Five pedigrees illustrating the segregation of copy number variant (CNV) alleles. Figure S3. The relationship of increased HAS2 and HAS2as gene expression with increased CNV_16.1 copy number holds with droplet digital PCR (ddPCR) CNV measures. (DOCX 1628 kb) [file 12864_2016_2619_MOESM2_ESM.docx]

**Olsson et al., 2015 “Absolute quantification reveals the stable transmission of a high copy number variant linked to autoinflammatory disease”**

**Supplementary information**

**Table S2**. Phenotypic overlap observed within the genetic test cohort

| Phenotype | Abbreviation^1^ | *n* |
| --- | --- | --- |
| Single | F | 28 |
|  | Ar | 3 |
|  | V | 4 |
|  | Am | 13 |
| Double | F.Ar | 31 |
|  | F.V | 3 |
|  | F.O | 7 |
|  | F.Am | 7 |
|  | Ar.Am | 3 |
|  | V.O | 2 |
|  | O.Am | 1 |
|  | V.Am | 4 |
| Triple | F.Ar.O | 7 |
|  | F.Ar.Am | 3 |
|  | F.V.O | 3 |
|  | F.V.Am | 21 |
|  | V.O.Am | 1 |
| Quadruple | F.Ar.V.O | 5 |
|  | F.Ar.O.Am | 2 |
|  | F.V.O.Am | 1 |

^1^Fever (F), Arthritis (Ar), Vesicular Hyaluronosis (V), Otitis (O), Amyloidosis (Am). “F.Ar” would indicate an individual with both Fever and Arthritis.

**Table S3.** Primers and Probes used in analysis

| Assay | Forward Primer 5’-3’ | Reverse Primer 5’-3’ | Probe 5’-3’ |
| --- | --- | --- | --- |
| CNV-East | AAC AGC AAA CGC GGA ATG AT | GAC TTC TCC GAA TCT TAA CTG CTA ATG | FAM - TGT CCT TCT TAG AAA TTC AA |
| CNV-759 | GAA AAT TCA AAC CAC CCT CAG TAT C | ACA GCC ACA ATC TTC AGG GTA GA | FAM - TAT CCT CCA TAG GAC ACA AT |
| CNV-E | TTA TGT TTT GCT GCC CTA GTC AGA | ACC TGG CAC CTG AGC AAC TT | FAM - CTG CTG AAA AGA CAC ATG T |
| C7orf28b Housekeeper | CAA CAC AGG TTG ACC AAG GA | TTG TGC AGG ATC AGA GCA TC | VIC - TGC CAT TTG TGT GCA TCC CCA |

**
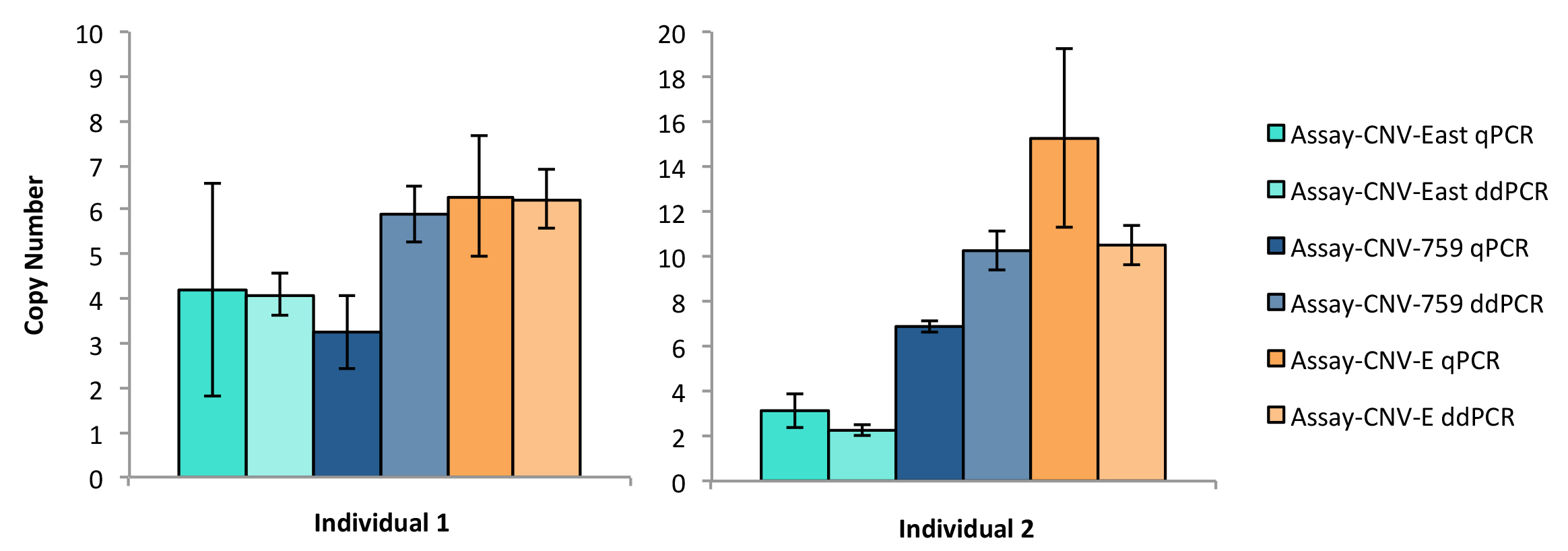
**

**Figure S1.** Illustrative plots for two individuals showing the results obtained for each assay and methodology. Inconsistencies can be seen between the qPCR results for CNV-16. (i.e. Assay-CNV-759 qPCR versus Assay-CNV-E qPCR) not observed for ddPCR. Large errors values are observed in the qPCR data adding uncertainty to the true copy number value.


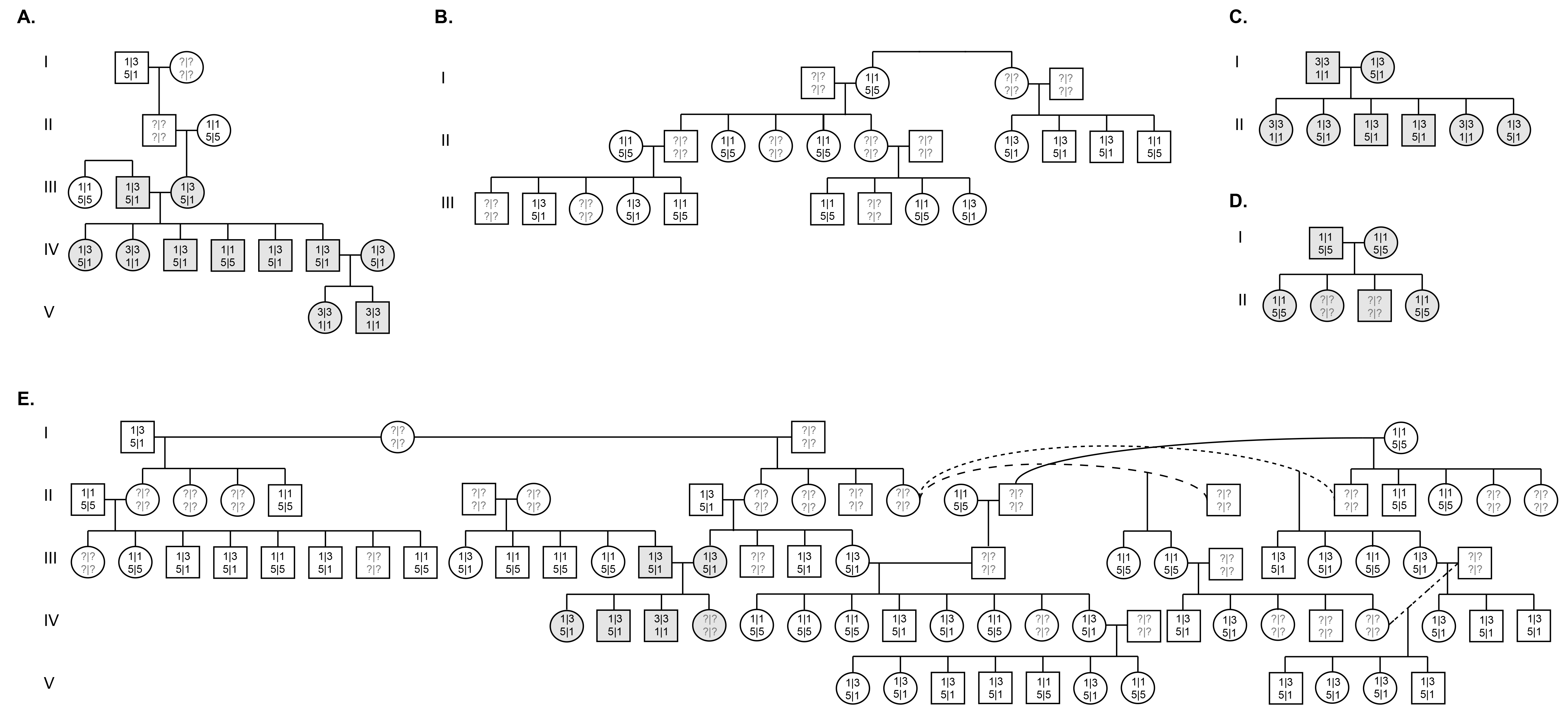


**Figure S2**. Five pedigrees illustrating the segregation of copy number variant (CNV) alleles. Figures **A**, **B** and **E** are multigenerational, whilst **C** and **D** show single matings. CNV results are coded to reflect the number of alleles per chromosome. For example, in **A** the male in generation has the genotyping result of CNV_14.3 (copy number: 4; alleles: 1|3) written above CNV_16.1 (copy number: 6; alleles: 5|1). Where genetic samples were not available genotypes are indicated as ?|?. Cases where it was possible to genotype parents and offspring are highlighted in grey. The grey section of **A** is Figure 2 in the main text.


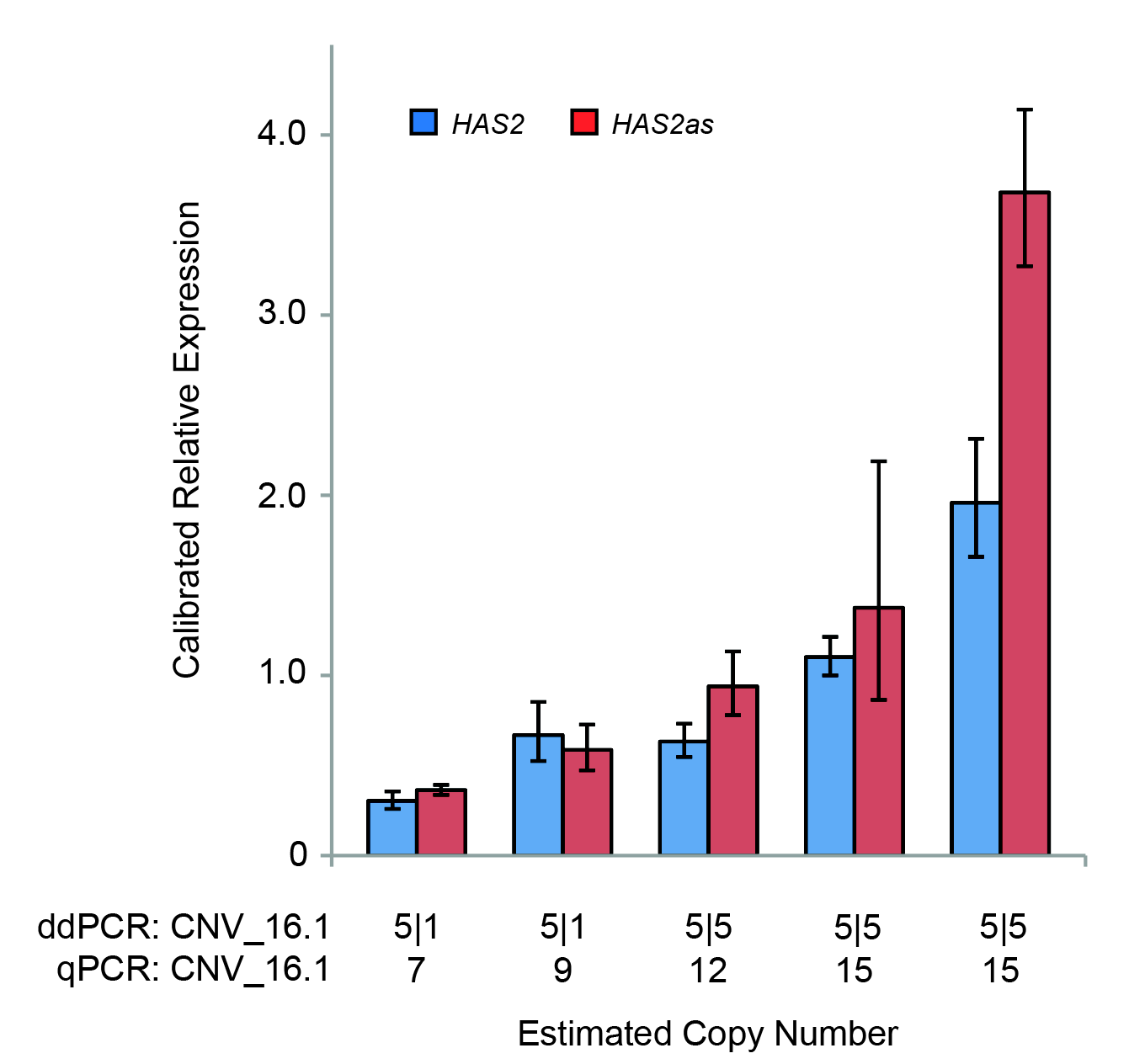


**Figure S3.** The relationship of increased *HAS2* and *HAS2as* gene expression with increased CNV_16.1 copy number holds with droplet digital PCR (ddPCR) CNV measures. Dermal fibroblast expression levels were taken from Olsson *et al*., 2011 and were normalised with the sample the lowest copy number (CNV = 5 measured with qPCR in Olsson et al., 2011; CNV_16.1 = 6 copies measured with ddPCR in this study). For each sample the ddPCR for this study is given per chromosome for CNV_16.1 (e.g. 6 copies; 5|1), followed by the qPCR CNV_16.1 estimation from Olsson *et al*., 2011.
